# N-Myc promotes therapeutic resistance development of neuroendocrine prostate cancer by differentially regulating miR-421/ATM pathway

**DOI:** 10.1186/s12943-019-0941-2

**Published:** 2019-01-18

**Authors:** Yu Yin, Lingfan Xu, Yan Chang, Tao Zeng, Xufeng Chen, Aifeng Wang, Jeff Groth, Wen-Chi Foo, Chaozhao Liang, Hailiang Hu, Jiaoti Huang

**Affiliations:** 1 Department of Urology, First Affilated Hospital of Anhui Medical University, Hefei, 230022 China; 2 Department of Pathology, Duke Unversity School of Medicine, DUMC box 103864, 905 S. Lasalle Street, Durham, NC 27710 USA; 3 Department of Pathology, Anhui Medical University, Hefei, 230032 China; 4 Institute of Clinical Pharmacology, Anhui Medical University, Hefei, China; 5 Department of Urology, Jiangxi Province People’s Hospital, Nanchang, China; 6 Duke Cancer Institute, Duke University School of Medicine, Durham, NC 27710 USA; 7 Department of Pharmacology and Cancer Biology, Duke University School of Medicine, Durham, NC 27710 USA

**Keywords:** Neuroendocrine, ATM, Senescence, ATM inhibitor, EZH2

## Abstract

**Background:**

*MYCN* amplification or N-Myc overexpression is found in approximately 40% NEPC and up to 20% CRPC patients. N-Myc has been demonstrated to drive disease progression and hormonal therapeutic resistance of NEPC/CRPC. Here, we aim to identify the molecular mechanisms underlying the N-Myc-driven therapeutic resistance and provide new therapeutic targets for those N-Myc overexpressed NEPC/CRPC.

**Methods:**

N-Myc overexpressing stable cell lines for LNCaP and C4–2 were generated by lentivirus infection. ADT-induced senescence was measured by SA-β-gal staining in LNCaP cells in vitro and in LNCaP xenograft tumors in vivo. Migration, cell proliferation and colony formation assays were used to measure the cellular response after overexpressing N-Myc or perturbing the miR-421/ATM pathway. CRISPR-Cas9 was used to knock out ATM in C4–2 cells and MTS cell viability assay was used to evaluate the drug sensitivity of N-Myc overexpressing C4–2 cells in response to Enzalutamide and ATM inhibitor Ku60019 respectively or in combination.

**Results:**

N-Myc overexpression suppressed ATM expression through upregulating miR-421 in LNCaP cells. This suppression alleviated the ADT-induced senescence in vitro and in vivo. Surprisingly, N-Myc overexpression upregulated ATM expression in C4–2 cells and this upregulation promoted migration and invasion of prostate cancer cells. Further, the N-Myc-induced ATM upregulation in C4–2 cells rendered the cells resistance to Enzalutamide, and inhibition of ATM by CRISPR-Cas9 knockout or ATM inhibitor Ku60019 re-sensitized them to Enzalutamide.

**Conclusions:**

N-Myc differentially regulating miR-421/ATM pathway contributes to ADT resistance and Enzalutamide resistance development respectively. Combination treatment with ATM inhibitor re-sensitizes N-Myc overexpressed CRPC cells to Enzalutamide. Our findings would offer a potential combination therapeutic strategy using ATM kinase inhibitor and Enzalutamide for the treatment of a subset of mCRPC with N-Myc overexpression that accounts for up to 20% CRPC patients.

**Electronic supplementary material:**

The online version of this article (10.1186/s12943-019-0941-2) contains supplementary material, which is available to authorized users.

## Introduction

Neuroendocrine prostate cancer (NEPC) represents the lethal endpoint stage of prostate cancer and is more commonly thought to emerge from prostate cancer adenocarcinoma (PCA) after hormonal therapy [6, 25, 32]. NEPC has a unique gene expression profile that is distinguishable from PCA [5, 22] and CRPC with neuroendocrine morphology (CRPC-NE) harbors a different genetic mutation landscape and epigenetic methylation status from CRPC with adenocarcinoma phenotype (CRPC-Ade) [4]. Oncogene amplification (such as *MYCN and AURKA*) and/or tumor suppressor mutation or deletion (e.g. *TP53* and *RB1*) can lead to the progression from PCA to NEPC [5, 35]. Other proteins that include PEG10, SRRM4, SOX2 and BRN2 have been shown to play an important role in the development of NEPC [2, 7, 24, 29].

Although *MYCN* gene amplification and/or N-Myc oncoprotein overexpression is found in ~ 40% NEPC [5] and up to 20% CRPC without neuroendocrine phenotype [13], they are also found in ~ 5% PCA [5, 28], suggesting that these amplification events can arise early before hormonal therapy. Two recent studies have firmly established N-Myc as an oncogenic driver for NEPC tumorigenesis [13, 22]. *Lee* et al. took advantage of their tissue recombination system to demonstrate that N-Myc overexpression in human prostate epithelial cells, together with activated AKT (Myr-Akt), can initiate both PCA and NEPC tumorigenesis and the resulted N-Myc/Myr-Akt tumors are castration resistant and metastatic with low level of AR expression [22]. *Dardenne* et al. utilized transgenic animal models to show that N-Myc can cooperate with EZH2 to drive the progression from CRPC-Ade to CRPC-NE and the co-operation confers the resistance to the newer generation of AR-targeted therapies including Enzalutamide [13]. N-Myc overexpression, whatever in PCA or in CPRC stage, shuts down AR signaling that is required for prostate cancer growth, and as a consequence should benefit the N-Myc overexpressed prostate tumors to AR-targeted therapies. However, the N-Myc overexpressed prostate tumors are resistant to AR-targeted therapies, including ADT and Enzalutamide, indicating that N-Myc re-establishes other AR-independent pro-survival mechanisms/pathways to drive the disease progression and therapeutic resistance development. Unfortunately, these N-Myc-induced new pro-survival mechanisms/pathways remain largely unknown.

In this study, we identified an N-Myc-regulated DNA damage response (DDR) pathway (N-Myc/miR-421/ATM) that contributes to the N-Myc-driven disease progression and hormonal therapeutic resistance. We further showed that inhibition of ATM by CRISPR knockout or ATM kinase inhibitor re-sensitized N-Myc overexpressed CRPC cells to Enzalutamide.

## Results

### N-Myc overexpression confers LNCaP cells the resistance to ADT and C4–2 cells the resistance to Enzalutamide

**Fig. 1:**
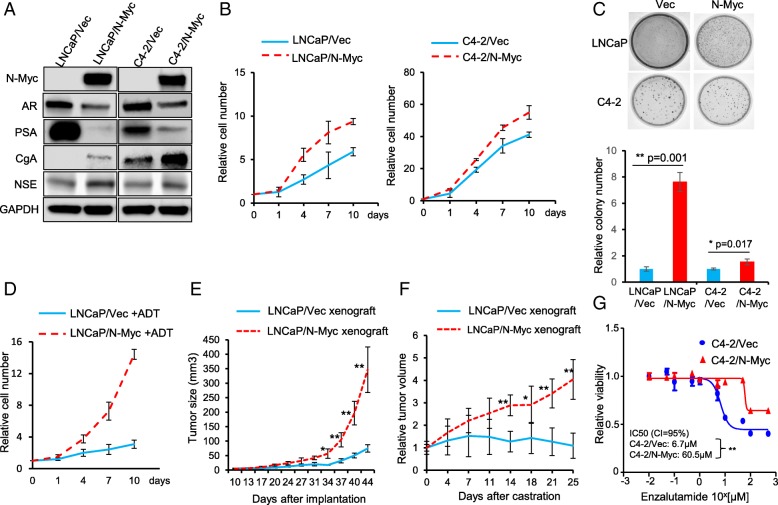
N-Myc confers LNCaP cells ADT resistance and C4–2 cells Enzalutamide resistance. **a** Immunoblots to show the decreased AR and PSA expression but increased expression of neuroendocrine markers, CgA and NSE, in both N-Myc overexpressing LNCaP and C4–2 cells. GAPDH was used as a loading control. **b** Growth curve for LNCaP/Vec vs. LNCaP/N-Myc and C4–2/Vec vs. C4–2/N-Myc cells cultured in regular medium and cell number was counted at the indicated time points. All the numbers were normalized to day 0. **c** Overexpression of N-Myc increased colony formation in both LNCaP and C4–2 cell lines but had a more profound effect on LNCaP cells. Upper panels showed representative images and lower panels were quantifications. **d** Growth curve for LNCaP/Vec and LNCaP/N-Myc cells cultured in charcoal-stripped medium (ADT) and cell numbers was counted at the indicated time points. All the numbers were normalized to day 0. **e** LNCaP/Vec and LNCaP/N-Myc cells were injected into the flanks of nude mice. Tumor volume was measured twice a week at indicated time points (*n* = 4 for each group). **f** Castration was initiated when tumor volume reached ~ 200mm^3^ and set as day 0. Tumor volume was measured twice a week and relative volume was reported (*n* = 4 for each group). **g** Dose response of Enzalutamide at 72 h using MTS cell viability assay for C4–2/Vec and C4–2/N-Myc cells. *p < 0.05, **p < 0.005

To recapitulate the N-Myc-driven therapeutic resistance of prostate cancer to ADT and Enzalutamide in vitro, we generated N-Myc overexpressing stable cell lines for RWPE-1, LNCaP and C4–2, which represent normal, androgen-responsive PCA and androgen-independent CRPC, by lentivirus infection. N-Myc overexpression in LNCaP and C4–2 cells resulted in decreased expression of AR and PSA but increased expression of neuroendocrine cell markers CgA and NSE (Fig. 1a), whereas N-Myc overexpression in normal RWPE-1 prostate epithelial cells was unable to induce the molecular neuroendocrine phenotype (Additional file 1: Figure S1). This is consistent with a recent report that N-Myc needs to cooperate with activated AKT to drive the neuroendocrine prostate cancer progression from normal human prostate epithelial cells [22], and LNCaP and C4–2 cells already have active AKT due to PTEN deficiency in them. Although N-Myc can induce a molecular neuroendocrine phenotype for both LNCaP and C4–2 cells, N-Myc overexpression dramatically increased the cell proliferation for LNCaP cells whereas had a marginal stimulatory effect on C4–2 cell proliferation as shown by growth curve and colony formation capacity (Fig. 1 b &c), suggesting that N-Myc overexpression has a more profound effect on cell proliferation of androgen-responsive prostate cancer cells than that of CRPC cells.

We then examined whether N-Myc overexpression leads to the resistance of LNCaP cells to ADT and the resistance of C4–2 cells to Enzalutamide. As expected, ADT treatment (culture cells in charcoal striped serum containing media) significantly decreased the proliferation of control LNCaP/Vec cells and N-Myc overexpression overcame the ADT-induced growth arrest and promoted the proliferation of LNCaP cells in a similar growth rate to that of cells in the presence of androgen (Fig. 1d). In agreement with the in vitro results, N-Myc overexpression remarkably promoted the LNCaP xenograft tumor growth without castration surgery (Fig. 1e), and abrogated the ADT (castration surgery)-induced growth arrest of LNCaP xenograft tumors (Fig. 1f). In a similar way, N-Myc overexpression led C4–2 cells to become more resistant to Enzalutamide (IC50 = 60.5 μM for C4–2/N-Myc cells compared to IC50 = 6.7 μM for C4–2/Vec cells) (Fig. 1g).

Taken together, N-Myc overexpression induced a molecular neuroendocrine phenotype in both LNCaP and C4–2 cells, and suppressed AR expression and AR signaling (indicated by PSA expression). The ADT- and Enzalutamide-resistance caused by N-Myc overexpression strongly implicates that N-Myc activates other pro-survival pathways or mechanisms to sustain the cell proliferation and growth independently of AR.

### N-Myc overexpression alleviates ADT- induced senescence in LNCaP cells in vitro and xenograft tumors in vivo

**Fig. 2:**
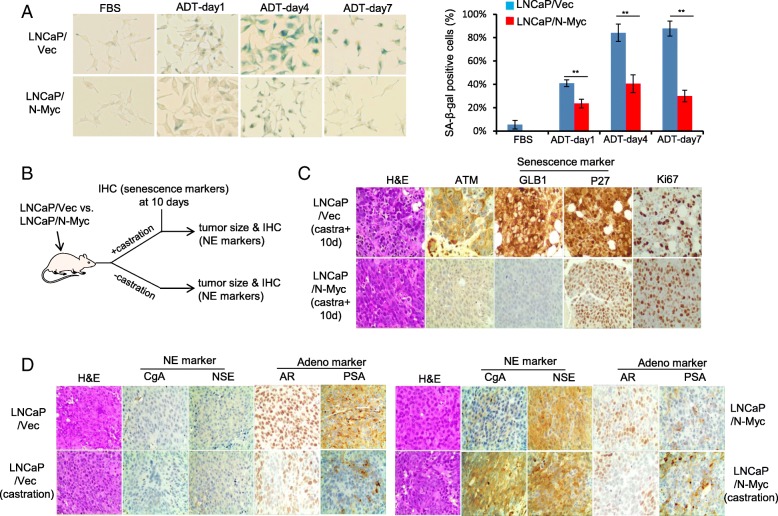
N-Myc overexpression alleviates ADIS in LNCaP cells in vitro *and* in vivo*.* **a** Representative images of senescence associated beta-galactosidase (SA-β-gal) stained LNCaP/Vec and LNCaP/N-Myc cells treated with ADT at indicated time points. Quantifications of SA-β-gal positively stained cells were presented in the right panels. **b** Schematic representing xenograft treatment cohorts and IHC staining at 10 days after castration or at the end of the experiments. **c** Immunohistochemistry staining of senescence markers (GLB1 and p27) and proliferation marker Ki67 for LNCaP/Vec and LNCaP/N-Myc xenograft tumors 10-day post castration treatment. **d** Immunohistochemistry staining of NE markers (CgA and NSE) and prostate adenocarcinoma markers (AR and PSA) for LNCaP/Vec and LNCaP/N-Myc xenograft tumors with or without castration treatment. Scale bar, 50-μm. ** p < 0.005

ADT is known to initially suppress tumor growth but eventually fails as the ADT resistant (castration resistant) tumors recur and develop with time. The resistant mechanisms have been mostly linked to AR-related aberrations, such as AR amplification, AR mutation, AR activation by promiscuous ligand and altered AR co-regulator expression [12, 37], which are usually identified in the late-stage castration resistant tumors. Actually, little is known about the earliest changes during ADT treatment. ADIS (ADT-induced senescence) is an early response of prostate adenocarcinoma to ADT [16] and evasion of ADIS has been demonstrated to promote the outgrowth of androgen-refractory prostate cancer [9]. To test whether N-Myc overexpression leads to the ADT resistance of LNCaP cells by escaping the ADIS, we performed an ADT treatment on LNCaP/Vec and LNCaP/N-Myc cells and ADIS was measured by SA-β-gal staining. The percentage of SA-β-gal positive senescent LNCaP/Vec cells increased over the ADT treatment time whereas N-Myc overexpression significantly reduced the percentage of ADT-induced SA-β-gal positive senescent cells (Fig. 2a). It is known that radiotherapy is a treatment option for early prostate cancer and irradiation (IR) can induce senescence as well [14]. N-Myc overexpression functioned in a similar way to that of ADT to reduce the percentage of IR-induced SA-β-gal positive senescent cells (Additional file 2: Figure S2).

To confirm whether N-Myc overexpression alleviates the ADIS in vivo, we injected LNCaP/vec and LNCaP/N-Myc cells subcutaneously into male SCID/NSG mice and conducted a castration surgery when xenograft tumors reached about 200mm^3^. 10 days after castration treatment, xenograft tumors were obtained and subject to immunohistochemistry (IHC) staining (Fig. 2b). The staining signals of senescence marker GLB1 (β-galactosidase) and cell cycle G1 phase marker p27 were much weaker in N-Myc overexpressing xenografts while the staining signal of cell proliferation marker Ki67 was stronger in the N-Myc overexpressing xenograft (Fig. 2c), indicating that N-Myc overexpression alleviates the ADT (castration treatment)-induced senescence (arrest in G1 phase) in vivo.

We further analyzed the IHC staining of paired tumor xenografts obtained at the end of the experiment (Fig. 2b). Without castration treatment, N-Myc overexpressing LNCaP xenografts displayed more neuroendocrine prostate cancer phenotypes (indicated by CgA and NSE staining) than control LNCaP xenografts which showed more PCA phenotypes (indicated by AR and PSA staining) (Fig. 2d). With castration treatment, control LNCaP xenografts exhibited less AR and PSA staining compared to non-castrated LNCaP xenografts, suggestive of treatment efficiency; while N-Myc overexpressing LNCaP xenografts showed more CgA and NSE staining compared to non-castrated N-Myc LNCaP xenografts (Fig. 2d), suggesting that N-Myc overexpression survives the ADT treatment and further drives the PCA progressing to NEPC.

### Downregulation of ATM by N-Myc via miR-421 mediates the ADIS alleviation

**Fig. 3:**
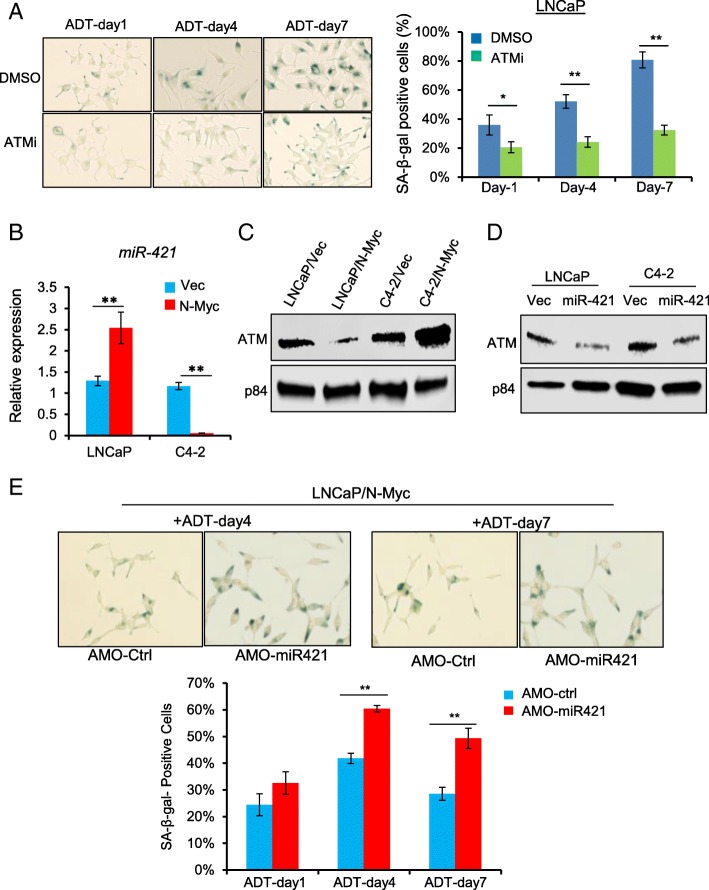
N-Myc-induced down-regulation of ATM via miR-421 mediates the ADIS alleviation in LNCaP cells. **a** ATM inhibitor Ku60019 (10 μM) decreased ADT-induced senescence. Left panels showed representative images at indicated time points. Quantifications of SA-β-gal positively stained cells were indicated in the right panel. **b** RT-qPCR to show N-Myc overexpression increased miR-421 expression LNCaP cells but decreased miR-421 expression in C4–2 cells. **c** Immunoblots to show the decreased protein level of ATM in N-Myc overexpressing LNCaP cells but increased protein level of ATM in N-Myc overexpressing C4–2 cells. P84 is a loading control. **d** Transient transfection of miR-421 mimics leads to ATM suppression in both LNCaP and C4–2 cells. **e** Antisense morpholino oligonucleotide (AMO-miR421) increased the percentage of positive SA-β-gal staining cells in N-Myc overexpressed LNCaP cells upon ADT treatment. Upper panels showed representative images at day 4 and day 7. Quantifications of SA-β-gal positively stained cells were indicated in the lower panel. *p < 0.05; **p < 0.005

ATM kinase is well known for its essential role in oncogene-induced senescence [3, 15, 26]. To examine whether ATM is involved in ADIS, we treated LNCaP cells with ATM kinase specific inhibitor KU-60019 together with the ADT treatment and found that KU-60019 significantly reduced the percentage of ADT-induced SA-β-gal positive senescent cells (Fig. 3a), indicating the involvement of ATM in ADIS. This is also consistent with a recent report that ATM inhibitor KU-60019 is identified by a high throughput screening as an effective agent to alleviate senescence [21]

We have previously demonstrated that N-Myc overexpression can down-regulate ATM expression via a human microRNA, miR-421, in *MYCN*-amplified neuroblastoma cells [19]. It prompted us to see whether N-Myc overexpression overcomes the ADIS by modulating miR-421/ATM pathway in prostate cancer cells. As shown in Fig. 3 b &c, N-Myc overexpression up-regulated miR-421 expression and consequently down-regulated ATM protein expression in LNCaP cells, in consistent with our previous results found in neuroblastoma cells. We have used antisense morpholino oligonucleotide (AMO-miR421) to modulate miR-421 and ATM expression in neuroblastoma cells [19]. Treating LNCaP/N-Myc cells with AMO-miR421, which inhibited miR-421 expression and up-regulated ATM expression, resulted in the increased percentage of ADT-induced SA-β-gal positive senescent cells (Fig. 3e). Similarly, inhibition of miR-421 by AMO-miR421 in LNCaP/N-Myc cells led to the increased percentage of IR-induced SA-β-gal positive senescent cells as well (Additional file 2: Figure S2). Taken together, ATM is involved in ADIS and its downregulation mediates the N-Myc overexpression-induced senescence alleviation through miR-421.

### Upregulation of ATM expression by N-Myc in C4–2 cells contributes to Enzalutamide resistance development

To our surprise, N-Myc overexpression in castration resistant C4–2 cells resulted in up-regulation of ATM expression instead of down-regulation observed in LNCaP cells (Fig. 3c). Interestingly, miR-421 expression was significantly decreased in N-Myc overexpressing C4–2 cells (Fig. 3b), reversely correlating with the upregulation of ATM expression. Actually, miR-421 overexpression by transient transfection suppressed ATM protein expression in both LNCaP and C4–2 cells (Fig. 3d), suggesting that miR-421 negatively regulates ATM expression in both androgen-dependent and -independent prostate cancer cells, and N-Myc -induced ATM up-regulation in C4–2 cells is caused by down-regulation of miR-421 and de-repression of ATM expression.

**Fig. 4:**
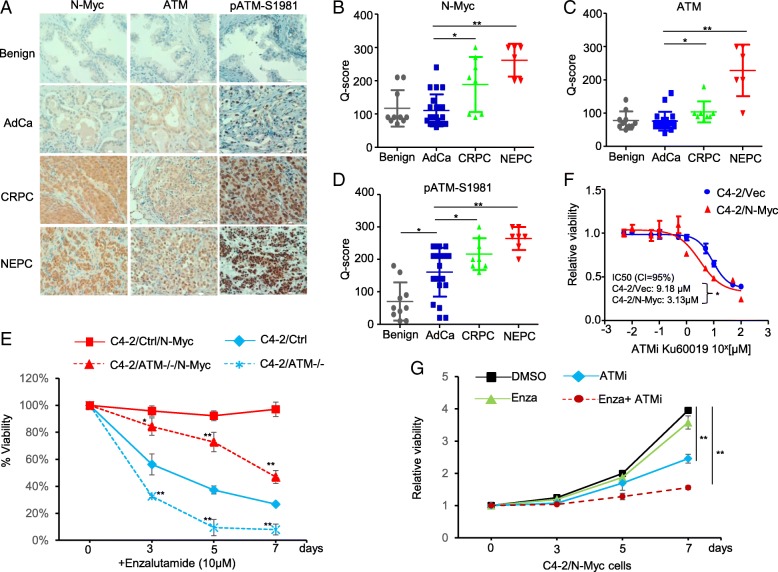
Upregulation of ATM by N-Myc contributes to Enzalutamide resistance of C4–2 cells. **a** Immunohistochemistry staining of N-Myc, ATM and phospho-ATM at serine 1981 (p-ATMS1981) in human benign prostate, adenocarcinoma prostate cancer (AdCa), CRPC and NEPC tissues. **b**-**d** Quantification of N-Myc, ATM and p-ATMS1981 IHC staining from (**a**). **e** Cell viability of different C4–2 cells treated with 10 μM Enzalutamide over a time course. C4–2/Ctrl and C4–2/ATM−/− represents CRISPR control and CRISPR ATM knockout while C4–2/Ctrl/N-Myc and C4–2/ATM−/−/N-Myc are the cells with N-Myc overexpression introduced by lentivirus infection. **f** Dose response of ATM inhibitor Ku60019 at 72 h using MTS cell viability assay for C4–2/Vec and C4–2/N-Myc cells. **g** Cell viability of C4–2/N-Myc treated with DMSO, Enzalutamide (10 μM), ATMi Ku60019 (2 μM) and in combination (Enza (10 μM) + ATMi (2 μM)) over a time course. *p < 0.05, **p < 0.005

The up-regulation of ATM by N-Myc overexpression in C4–2 cells is further confirmed by IHC staining of N-Myc and ATM in different stages of human prostate tumor tissues. N-Myc expression was increased with the disease progression with the highest level in NEPC tissue (Fig. 4 a&b). ATM positive nuclear staining was similar to N-Myc expression pattern with the strongest signals in NEPC tissues and positively correlates with N-Myc expression (Fig. 4 a&c). Interestingly, the signal of phospho-ATM (indicative of active ATM) staining was also the strongest in NEPC tissues (Fig. 4a&d), suggesting that many ATM-mediated phosphorylation events occur in NEPC that may mediate the N-Myc-driven disease progression and therapeutic resistance development.

We have demonstrated that N-Myc overexpression in C4–2 cells resulted in a significantly enhanced in vitro resistance to Enzalutamide (Fig. 1g). To investigate whether ATM upregulation in C4–2/N-Myc cells mediates the Enzalutamide resistance, we used CRISPR-Cas9 lentiviral deliver system to knock out ATM in C4–2 cells, and subjected them to Enzalutamide treatment. ATM knockout further enhanced the sensitivity of C4–2 cells to Enzalutamide (Fig. 4e). Introducing N-Myc into the C4–2/CRISPR-control cells led to the Enzalutamide resistance as expected, and the ATM knockout re-sensitized the N-Myc overexpressing C4–2 cells to Enzalutamide (Fig. 4e). This observation provides a therapeutic opportunity for N-Myc overexpressed CRPC by pharmacological inhibition of ATM. Indeed, C4–2/N-Myc showed increased in vitro sensitivity to ATM kinase specific inhibitor Ku60019 with IC50 = 3.13 μM compared to control C4–2/Vec cells with IC50 = 9.18 μM (Fig. 4f). Furthermore, Ku60019 can re-sensitize C4–2/N-Myc cells to Enzalutamide in a combination treatment (Fig. 4g). Collectively, both ATM knockout and pharmacological inhibition of ATM enable C4–2/N-Myc cells become sensitive to Enzalutamide, suggesting a potential combination therapy with ATM inhibitor and Enzalutamide for N-Myc overexpressed CRPC that accounts for up to 20% CRPC patients.

### N-Myc promotes migration/invasion by up-regulating ATM expression via miR-421

**Fig. 5:**
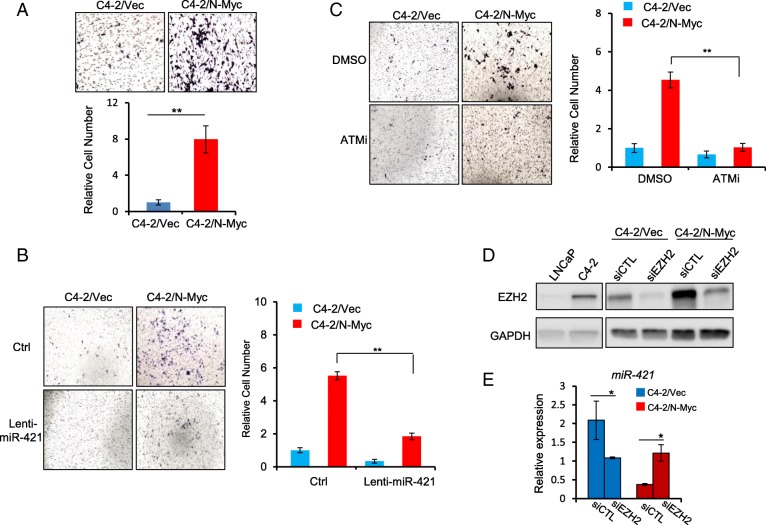
N-Myc-induced up-regulation of ATM promotes migration/invasion of C4–2 cells. **a** Representative images of migration/invasion capacity for C4–2/Vec and C4–2/N-Myc cells passing through a Trans-well barrier for 48 h. Bottom panel showed the quantification of relative migrated cell number. **b** and **c** representative images of C4–2/Vec and C4–2/N-Myc cells migration treated by either miR-421 overexpression by lentivirus infection or ATM inhibitor (10 μM), and corresponding quantifications. **d** Immunoblot to show endogenous EZH2 protein level of LNCaP and C4–2 cells and knockdown by siRNA (siEZH2) in C4–2/Vec and C4–2/N-Myc cells. GAPDH was used as a loading control. **e** RT-qPCR to show the increased level of miR-421 in C4–2/N-Myc cells instead of decreased after EZH2 siRNA knockdown. * *p* < 0.05, ** *p* < 0.005

NEPC and Enzalutamide-resistant CRPC are known to be more metastatic and ATM has been shown to promote tumor migration and invasion through its non-DNA repair functions [10, 34]. Based on the observation that N-Myc overexpression in C4–2 cells only marginally increased cell proliferation (~ 1.5 fold) (Fig. 1 b&c), we wanted to know whether the N-Myc-induced ATM upregulation in CRPC contributes to its invasive phenotype. We used trans-well migration assay to show that N-Myc overexpression in C4–2 cells dramatically enhanced cell migration and invasion (~ 4–8 fold) (Fig. 5a). Overexpression of miR-421 into C4–2/N-Myc cells by lentivirus infection suppressed ATM expression (Additional file 3: Figure S3) and abrogated the N-Myc-enhanced migration/invasion (Fig. 5b), suggesting that N-Myc-enhanced migration/invasion is mediated, at least partially, by miR-421/ATM pathway. Further, treatment of C4–2/N-Myc cells by ATM inhibitor KU60019 abrogated the N-Myc-increased migration as well (Fig. 5c), implicating that ATM inhibitor not only re-sensitize N-Myc overexpressed CRPC cells to Enzalutamide, but also prevent their metastasis.

We showed that N-Myc differentially regulated miR-421 expression in LNCaP and C4–2 cells (Fig. 3b), which is corroborated with a most recent report showing that N-Myc overexpression in LNCaP and 22RV1 (another CRPC cell line) can differentially affect the expression of a subset of genes (gene list is summarized in Additional file 4: Figure S4) and EZH2 has been shown to regulate some of these genes [13]. To test whether EZH2 switches the differential regulation of miR-421 by N-Myc, we first examined EZH2 protein level in LNCaP and C4–2 cells, and found that EZH2 expression was significantly increased in C4–2 cells (Fig. 5d). We then knocked down EZH2 with siRNA in C4–2/N-Myc cells and found that miR-421 expression was up-regulated instead of down-regulated (Fig. 5e), supporting our hypothesis that EZH2 cooperates with N-Myc to switch the upregulation of miR-421 in LNCaP cells to the downregulation of miR-421 in C4–2 cells. It will be interesting to see whether this is a general mechanism that N-Myc differentially regulates its target genes in a context-dependent manner and EZH2 works together with N-Myc to make this switch.

## Discussion

**Fig. 6:**
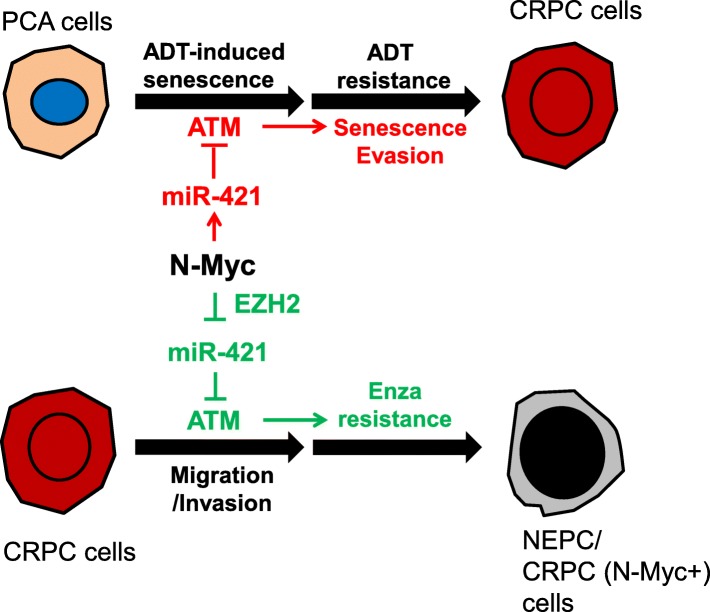
A working model: N-Myc differentially regulating miR-421/ATM pathway contributes to ADT resistance and Enzalutamide resistance development of prostate cancer. N-Myc overexpression, if occurs in androgen-responsive stage (PCA cells), can overcome the ADT-induced senescence by downregulating ATM expression via miR-421 and promotes the outgrowth of ADT-resistant cancer cells. In contrast, N-Myc overexpression, if occurs in androgen-independent CRPC stage, up-regulates ATM expression by cooperating with EZH2 to suppress miR-421 expression and then de-repress its suppression on ATM. The N-Myc-induced upregulation of ATM contributes to the Enzalutamide resistance development and promotes the migration ability of CRPC cells

The clinical observations that *MYCN* amplification and/or N-Myc overexpression is found in both NEPC and PCA [5, 28], together with the mechanistic studies showing that N-Myc suppresses AR signaling[13, 22], suggest that 1) *MYCN* amplification event can arise early in PCA before hormonal therapy; 2) N-Myc overexpression activates other AR-independent pro-survival mechanisms/pathways to allow the cells with N-Myc overexpression to repopulate the tumor after hormonal therapy, and therefore drives the resistance development to AR-targeted therapies. In this study, we demonstrated that N-Myc confers prostate cancer cells the ADT-resistance and Enzalutamide-resistance by differentially regulating ATM pathways. N-Myc overexpression in androgen-responsive PCA cells alleviates the ADIS by down-regulating ATM expression via miR-421 that consequently promotes the outgrowth of N-Myc-overexpressed cells from the ADT-induced proliferation-arrested cell subpopulation and progresses to CRPC (Fig. 6). In contrast, N-Myc overexpression in CRPC cells collaborates with EZH2 to up-regulate ATM expression through de-repressing the miR-421-induced ATM suppression. The N-Myc induced upregulation of ATM promotes the Enzalutamide resistance development as well as the migration/invasion of CRPC cells (Fig. 6). Although N-Myc differentially regulates miR-421/ATM pathway in different stages of prostate cancer, the biological consequence of both regulations is to facilitate the progression of NEPC from PCA and the development of therapeutic resistance. This might be a general mechanism for N-Myc to differentially regulate its target genes in androgen-dependent and -independent prostate cancer and EZH2 or other protein factor works together with N-Myc to make this switch.

ATM is a multi-functional protein kinase that is best known as a master regulator of DNA damage response [33]. ATM phosphorylates hundreds of proteins to induce the DNA damage response that includes cell cycle checkpoints, DNA repair, senescence and apoptosis [11, 27]. Our results demonstrated that ATM is required for ADIS like its involvement in other stress-induced senescence such as OIS (oncogene-induced senescence). If *MYCN* amplification event occurs in the PCA stage (~ 5% PCA patients), ADT treatment would kill most of androgen-responsive cancer cells but still induce a proliferation-arresting status (senescence) in a significant subpopulation [1]. N-Myc overexpression antagonizes the ADIS by downregulating ATM and then facilitates the evasion of senescence and promotes the outgrowth ADT-resistant cancer cells. If *MYCN* amplification and/or N-Myc overexpression occurs or keeps in CRPC stage (up to ~ 20% CRPC patients), the upregulation of ATM by N-Myc promotes the migration/invasion of CRPC cells and mediates, at least partially, the Enzalutamide resistance development. The precise mechanisms that how ATM regulates the Enzalutamide resistance need to be further characterized, but several studies suggest the link of ATM to AR signaling: 1) AR can induce TOP2B-mediated double strand break at its transcription sites and ATM is recruited to the DSB for repair and transcription [18]; 2) The telomere dysfunction caused by AR-inactivation in prostate cancer requires ATM activation to maintain the telomere, and therefore ATM inhibition potentiates the death of prostate cancer treated with AR antagonists [31]; 3) ATM can phosphorylate EZH2 and modulate its stability in neurons [23]. Given the strong staining of active ATM (pATM-S1981) in NEPC tissues (Fig. 4a) and high levels of EZH2 proteins in CRPC/NEPC tumors [13, 36], it is highly likely that ATM modulates EZH2 protein stability and activity in NEPC/CRPC and therefore affects the AR signaling and therapeutic resistance.

N-Myc, like c-Myc, is an undruggable target *per se*. Fortunately, *MYCN* amplification is found to co-occur with *AURKA* (encoding Auror-A kinase) gene amplification in NEPC [5, 28], in a similar way to that in neuroblastoma. Aurora-A kinase inhibitors have been shown to effectively target *MYCN* amplified neuroblastoma by destabilizing N-Myc expression [8, 17], which is also the basis for exploring Aurora-A kinase inhibitors for the treatment of NEPC or N-Myc overexpressed CRPC (clinical trial #NCT01799278). Indeed, Aurora-A kinase inhibitors have been shown to reduce N-Myc-induced NEPC tumor burden [5, 22]. A most recent study showed that N-Myc can transcriptionally activate DNA damage response proteins including PARP1 and PARP2, and combination treatment of CRPC and NEPC cells with Aurora-A kinase inhibitors and PARP inhibitor Olaparib results in significant suppression of tumor growth in preclinical models [38]. We demonstrated here that N-Myc upregulated ATM expression through a post-transcriptional microRNA regulatory mechanism, and this upregulation resulted in N-Myc overexpressing C4–2 cells more sensitive to ATM kinase inhibitor. Targeting ATM kinase by CRISPR/Cas9 knockout or ATM inhibitor re-sensitizes the N-Myc overexpressing C4–2 cells to Enzalutamide. These findings suggest a potential combination therapy for N-Myc overexpressed CRPC/NEPC with Enzalutamide and ATM kinase inhibitor.

## Conclusions

We identified an N-Myc-regulated downstream DDR pathway (N-Myc/miR-421/ATM) that contributes to the N-Myc-driven therapeutic resistance, including ADT resistance and Enzalutamide resistance. We demonstrated that inhibition of ATM by CRISPR knockout or ATM kinase inhibitor re-sensitized N-Myc overexpressed CRPC cells to Enzalutamide, which may suggest a new combination therapeutic strategy with ATM inhibitor and Enzalutamide for the N-Myc overexpressed CRPC patients.

## Materials and methods

### Cell culture, miRNA, AMO, ATM inhibitors and transfection

LNCaP and C4–2 cells (ATCC) were cultured in RPMI-1640 medium (ATCC) supplemented with 10% fetal bovine serum (FBS; Corning) or 10% charcoal-stripped fetal bovine serum (CSS; Denville) and 100 unit /ml penicillin/streptomycin (Gibco) at 37 °C in 21% oxygen /5% CO_2_ incubator. Precursor miRNA-421 and pre-miR-CTL were purchased from Applied Biosystems. SiRNA (siRNA-control and siRNA-EZH2) was purchased from Dharmacon. Antisense Morpholino Oligonucleotides (AMO) was custom synthesized based on the pre-miR-421 target sequence and conjugated with non-peptide chemicals (Vivo-morpholino) that are used to deliver AMO to cells (Gene-Tools). The sequence of AMO-miR-421 is 5’-GCGCCCAATTAATGTCTGTTGATGA-3′. A standard control vivo-AMO (AMO-scram) was purchased from Gene-Tools as well. Lentiviral vector with N-Myc was a gift from Dr. Owen Witte lab at UCLA [22]. ATM inhibitor KU60019 was purchased from Selleckchem. Transfections for miRNA precursors / siRNAs were perfomed with Lipofectamine RNAiMax (Invitrogen) according to the manufacturer’s protocols.

### Cell proliferation and senescence measurements

To determine the effects of N-Myc on cell proliferation of LNCaP and C4–2 in both FBS and CSS (charcoal striped serum) containing media, 1 × 10^4^ cells were seeded in 6-well plate. Cell counts were carried out in triplicate every 72 h using a hemocytometer. FBS or CCS media was refreshed every 3 days. To measure senescence, 1 × 10^4^ LNCaP cells were seeded in 6-well plates with CCS media and cells were fixed and stained with senescence-associated beta-galactosidase activity (SA-β-gal) according to the manufacturer’s protocol (Senescence β-Galactosidase Staining kit, Cell Signaling) at the indicated time points. To quantify positive staining, > 100 cells were counted for each sample in several fields of view in order to obtain a standard deviation. All samples ran in three independent experiments.

### Quantitative real-time PCR

Total RNA was extracted using RNeasy mini kits (Qiagen). For miR-421 quantification, cDNA was prepared using qScript microRNA cDNA Synthesis Kit (Quanta Biosciences) per manufacturer’s protocol. Perfecta microRNA Assays (Quanta Biosciences) were used to quantitate mature miR-421 expression according to the provided protocol. RNU66 or U6 expression assay was used as an internal control for miR-421 expression. Quantitative real-time PCR (qRT-PCR) was performed on ABI 7500 (Applied Biosystems). All PCRs were performed in triplicates.

### Colony formation assay

1 × 10^4^ Cells were seeded into 6-cm dishes and grown for up to 14 days for colony formation. Cell culture media was refreshed every 3 days and colonies were fixed with cold methanol and then stained with 1% crystal violet. The number of colonies was counted and imaged with a VersaDoc Imaging System (BioRad).

### Nuclear protein extraction and western blot

To measure ATM protein expression, nuclear lysates were isolated with an NE-PER Nuclear and Cytoplasmic Extraction Reagents kit (Thermo) with protease and phosphatase inhibitor (Thermo Fisher) included. Total protein lysates were extracted by RIPA buffer (Sigma). Protein concentrations were measured using the Bradford reagent (Bio-rad). Equal amounts of protein lysate were loaded onto a 4–15% SDS/PAGE pre-cast TGX Stain-Free Gel (Bio-Rad) and subsequently transferred onto PVDF membrane (Bio-Rad) at 30 V at 4 °C overnight. Immunoblot analysis was performed with the following antibodies: rabbit-anti-ATM (Novus, NB#100–104), mouse-anti-N-MYC (Santa Cruz, #SC-53993), rabbit-anti-NSE (Millipore, # 2716128), rabbit-anti-PSA (Dako, # 20035991), mouse-anti-CgA (Millipore, # MAB5268), rabbit-anti-AR (Millipore, #06–680), mouse-anti-GAPDH (Abcam, #ab9484), rabbit-anti-p84 (Invitrogen, #PA5–27816), rabbit-anti-EZH2 (Cell Signaling Technology, #5246). Following incubation with the appropriate secondary horseradish antibodies (Bio-Rad), blots were developed using the Chemiluminescent Substrate kit (Thermo).

### Transwell assays

1 × 10^4^ cells in serum-free media were plated onto the upper chamber with 8 μm pores (Millipore), and 10% FBS media was placed in the lower chamber in 24-well plates. The chambers were coated with 5x BME Solution and 10xCoating Buffer (Trevigen). Migrated cells were stained with crystal violet after incubation for 48 h. To determine the effect of ATM inhibitor on cells’ migration, cells were pre-treated with 2 μM KU60019 for 48 h prior to trans-well procedure. Randomly selected fields were photographed and stained cells were counted and student t-test was used for statistical analysis.

### Tissue microarrays and immunohistochemical staining

Construction of tissue microarray (TMA) has been described previously [20, 30]. Briefly, specimens were reviewed and attributed to benign, adenocarcinoma, castration-resistant and small cell neuroendocrine based on pathological and clinical features. Representative areas were pre-marked in the paraffin-embedded blocks on the basis of the hematoxylin and eosin (H&E) staining for quality control and then transferred from donor blocks to recipient blocks to construct tissue microarray blocks. Immunohistochemical staining (IHC) was performed as described in a previous study [30]. In short, paraffin sections of 5 μm thickness were prepared from tissue microarrays or xenograft tumors. The sections were sequentially deparaffinization and re-hydrated with xylene through graded ethanol. Endogenous peroxidase activity was blocked in 3% H_2_O_2_ for 10 min and boiled in 10 mM citrate buffer (pH 6.0) for 10 min for heat-induced antigen retrieval. Rabbit polyclonal anti-N-Myc antibody (dilution 1:100, Proteintech, #10159–2-AP), rabbit polyclonal anti-ATM antibody (dilution 1:100, Novus, # nb100–104), mouse-monoclonal anti-GLB1 (dilution 1:100, Abcam, # ab55176), mouse-monoclonal anti-p27 (dilution 1:100, BD biosciences, #610242), rabbit-polyclonal anti-Ki-67 (dilution 1:300, Thermo Fisher, # RM-9106), rabbit-polyclonal anti-NSE (dilution 1:200, Abcam, # ab53025), rabbit-polyclonal anti-PSA (dilution 1:5000, Dako, # A0562), mouse-anti-CgA (dilution 1:500, Thermo Fisher, #PA5–32349), rabbit-anti-AR (dilution 1:300, Santa Cruz, # sc-816) were used as primary antibodies. Quantification of IHC staining using Q-Score followed the protocol as described in a previous study [30].

### Animal experiments

All animal work was conducted in accordance with the NIH Guidelines of Care and Use of Laboratory Animals and approved by Duke Institutional Animal Care and Use Committee (IACUC). Briefly, 1 × 10^7^ cells were resuspended in 100 μl of saline with 50% Matrigel (Corning, #354248) and injected subcutaneously into both sides of flank regions of 6-week-old male NSG mice (Jackson Lab). Castration was initiated when tumors reached ~ 200mm^3^. Tumor volume was measured twice a week with calipers and calculated by the formula: length x width^2^ / 2. Mice were sacrificed once their tumors reached an approximate size of 1000mm^3^ or an experimental endpoint. Tumors were excised and fixed in 4% paraformaldehyde solution for further embedding in paraffin. 5 μm paraffin sections were stained delicately using the primary antibodies.

### CRISPR/Cas9-mediated ATM knockout

To establish ATM knockout stable cell line, we used the CRIPR/Cas9 technology. Single guided RNA (sgRNA) sequences were generated by CRISPR design tool (crispr.mit.edu). sgRNA sequences for ATM are available upon request. Annealed double stranded sgRNA oligos were ligated to the lentiCRISPR vector 2 (deposited by Dr. Feng Zhang to Addgene, Camridge, MA) at a ribonucleoprotein complex which expressed both Cas9 and sgRNA. To produce infectious transgenic lentivirus, the transfer plasmid was transfected into A293T cells together with packaging plasmid and envelope plasmid. After medium changed and a brief incubation period, supernatant containing the virus was removed and centrifuged to concentrate virus. Subsequently, cells were infected with lentivirus and selected by puromycin (5 μg/ml) for more than 14 days. Population that showed no target protein expression was confirmed by western blot.

### Cell viability assays

The MTS assay (Biovision) was employed to determine cell viability following the manufacturer’s protocol. 1000/well cells were seeded in 96-well plate. 10 μl /well MTS Reagent was added into each well and incubated for 1 h at each time point. Absorbance was measured at 490 nm using SpectraMax M3 reader with SoftMax Pro 6 software for data acquisition and analysis. For cells proliferation assay, results of each group were normalized to Day 0. For IC50 determination of ATM inhibitor and Enzalutamide, cells were treated by a series of concentration for 72 h. IC50 value was calculated by GraphPad Prism software. All assays were performed in triplicate.

### Statistical analysis

Statistical analysis was performed using Student t test for cell proliferation, colony formation, senescence analysis, transwell migration and tumor xenografts. *P* values are denoted as follows: *, *p* < 0.05, **, *p* < 0.005.

## Additional files

Additional file 1: **Figure S1.** (A) Endogenous protein expression of AR, PSA, NSE and CgA in RWPE-1 cells with and without N-Myc overexpression. Immunoblot showed no significant difference between RWPE-1/vector and RWPE-1/N-Myc cells for these protein markers. GAPDH was used as a loading control. (B) Western blotting to show that ATM is not altered in N-Myc-overexpressed RWPE-1 cells, downregulated in N-Myc-overexpressed LNCaP and upregulated in N-Myc-overexpressed C4–2 cells. (PPTX 23818 kb)

Additional file 2: **Figure S2.** (A) Representative images of senescence associated beta-galactosidase (SA-β-gal) stained LNCaP/Vec and LNCaP/N-Myc cells treated with irradiation (2Gy) at indicated time points. Quantifications of SA-β-gal positively stained cells were presented in the bottom panels. (B) The percentage of IR-induced SA-beta-gal positive LNCaP/N-Myc cells was increased after treated by antisense morpholino oligonucleotide (AMO-miR-421). (PPTX 635 kb)

Additional file 3: **Figure S3.** Immunoblot showed that ATM expression was suppressed by overexpressing lentiviral miR-421 in both C4–2/vector and C4–2/N-Myc cells. p84 was used as a loading control. (PPTX 308 kb)

Additional file 4: **Figure S4.** N-Myc overexpression in LNCaP and 22RV1 cells differentially regulates the expression of the same target genes. A subset of gene list has been summarized in four different groups: upregulated in both LNCaP/N-Myc and 22RV1/N-Myc, upregulated in LNCaP/N-Myc but downregulated in 22RV1/N-Myc, downregulated in LNCaP/N-Myc but upregulated in 22RV1/N-Myc and both downregulated in LNCaP/N-Myc and 22RV1/N-Myc. (PPTX 68 kb)

## Abbreviations

ADIS: Androgen Deprivation Induced Senescence
ADT: Androgen Deprivation Therapy
AMO: Antisense Morpholino Oligonucleotides
CgA: Chromogranin A
CRISPR: Clustered Regularly Interspaced Short Palindromic Repeats
CRPC: Castration Resistant Prostate Cancer
IHC: Immunohistochemistry
MTS: 3-(4,5-dimethylthiazol-2-yl)-2,5-diphenyltetrazolium bromide
NEPC: Neuroendocrine Prostate Cancer
NSE: Neuron Specific Enolase
PCA: Prostate Cancer Adenocarcinoma
SA-β-gal: Senescence Associated beta Galactasidase
TMA: Tissue Microarray
