# Correction to: N-Myc promotes therapeutic resistance development of neuroendocrine prostate cancer by differentially regulating miR-421/ ATM pathway

**DOI:** 10.1186/s12943-019-1034-y

**Published:** 2019-06-19

**Authors:** Yu Yin, Lingfan Xu, Yan Chang, Tao Zeng, Xufeng Chen, Aifen Wang, Jeff Groth, Wen-Chi Foo, Chaozhao Liang, Hailiang Hu, Jiaoti Huang

**Affiliations:** 1 Department of Urology, First Affilated Hospital of Anhui Medical University, Hefei, 230022 China; 2 Department of Pathology, Duke Unversity School of Medicine, DUMC box 103864, 905 S. Lasalle Street, Durham, NC 27710 USA; 3 Department of Pathology, Anhui Medical University, Hefei, 230032 China; 4 Institute of Clinical Pharmacology, Anhui Medical University, Hefei, China; 5 Department of Urology, Jiangxi Province People’s Hospital, Nanchang, China; 6 Duke Cancer Institute, Duke University School of Medicine, Durham, NC 27710 USA; 7 Department of Pharmacology and Cancer Biology, Duke University School of Medicine, Durham, NC 27710 USA

**Correction to: Mol Cancer**

**https://doi.org/10.1186/s12943-019-0941-2**

Following publication of the original article [1], the authors reported that name that appeared in published online version is incorrect. Aifeng Wang should be Aifen Wang. Corrected name is provided in the author group section above.
