# Supplementary material for: N-Myc promotes therapeutic resistance development of neuroendocrine prostate cancer by differentially regulating miR-421/ATM pathway
Source: Mol Cancer. 2019 Jan 18;18:11. doi: 10.1186/s12943-019-0941-2 (PMC6337850; doi:10.1186/s12943-019-0941-2)
Supplement: Supplementary file 1 — Figure S1. (A) Endogenous protein expression of AR, PSA, NSE and CgA in RWPE-1 cells with and without N-Myc overexpression. Immunoblot showed no significant difference between RWPE-1/vector and RWPE-1/N-Myc cells for these protein markers. GAPDH was used as a loading control. (B) Western blotting to show that ATM is not altered in N-Myc-overexpressed RWPE-1 cells, downregulated in N-Myc-overexpressed LNCaP and upregulated in N-Myc-overexpressed C4–2 cells. (PPTX 23818 kb) [file 12943_2019_941_MOESM1_ESM.pptx]

## Slide 1
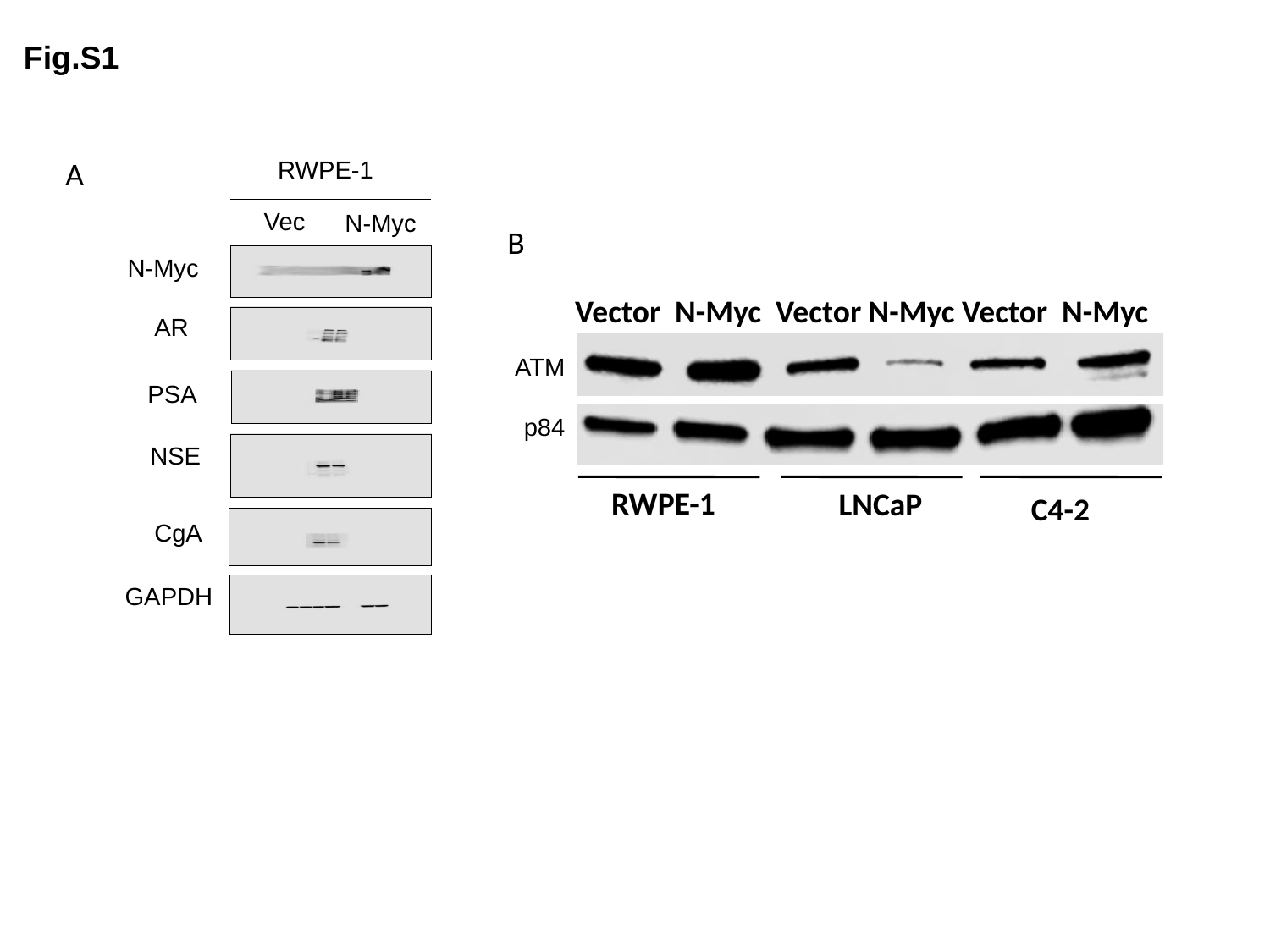

Fig.S1
A
RWPE-1
Vec
N-Myc
B
N-Myc
Vector N-Myc Vector N-Myc Vector N-Myc
AR
ATM
PSA
p84
NSE
RWPE-1
LNCaP
C4-2
CgA
GAPDH
