# Supplementary material for: N-Myc promotes therapeutic resistance development of neuroendocrine prostate cancer by differentially regulating miR-421/ATM pathway
Source: Mol Cancer. 2019 Jan 18;18:11. doi: 10.1186/s12943-019-0941-2 (PMC6337850; doi:10.1186/s12943-019-0941-2)
Supplement: Supplementary file 2 — Figure S2. (A) Representative images of senescence associated beta-galactosidase (SA-β-gal) stained LNCaP/Vec and LNCaP/N-Myc cells treated with irradiation (2Gy) at indicated time points. Quantifications of SA-β-gal positively stained cells were presented in the bottom panels. (B) The percentage of IR-induced SA-beta-gal positive LNCaP/N-Myc cells was increased after treated by antisense morpholino oligonucleotide (AMO-miR-421). (PPTX 635 kb) [file 12943_2019_941_MOESM2_ESM.pptx]

## Slide 1
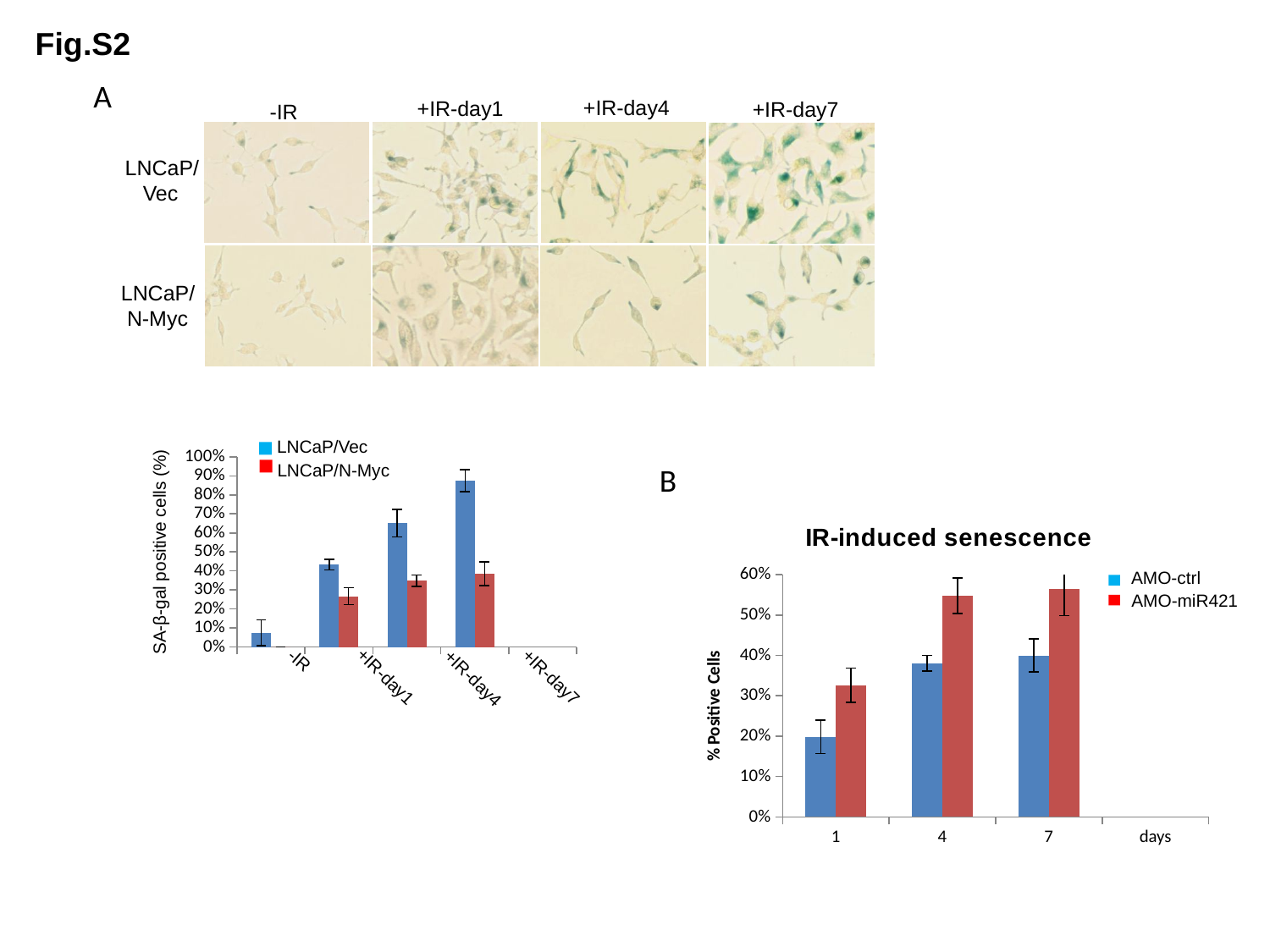

Fig.S2
A
+IR-day4
+IR-day1
+IR-day7
-IR
LNCaP/
 Vec
LNCaP/
 N-Myc
LNCaP/Vec
[unsupported chart]
LNCaP/N-Myc
B
### Chart: IR-induced senescence
| Category | MYCN | AMO(5uM) |
|---|---|---|
| 1 | 0.19830999066293184 | 0.32583087027914615 |
| 4 | 0.3803609116901042 | 0.5473522297808012 |
| 7 | 0.4000242120599034 | 0.5640541964041424 |
| days | None | None |SA-β-gal positive cells (%)
AMO-ctrl
AMO-miR421
-IR
+IR-day1
+IR-day7
+IR-day4
