# Supplementary figures and images for: N-Myc promotes therapeutic resistance development of neuroendocrine prostate cancer by differentially regulating miR-421/ATM pathway
Source: Mol Cancer. 2019 Jan 18;18:11. doi: 10.1186/s12943-019-0941-2 (PMC6337850; doi:10.1186/s12943-019-0941-2)

## Slide 1
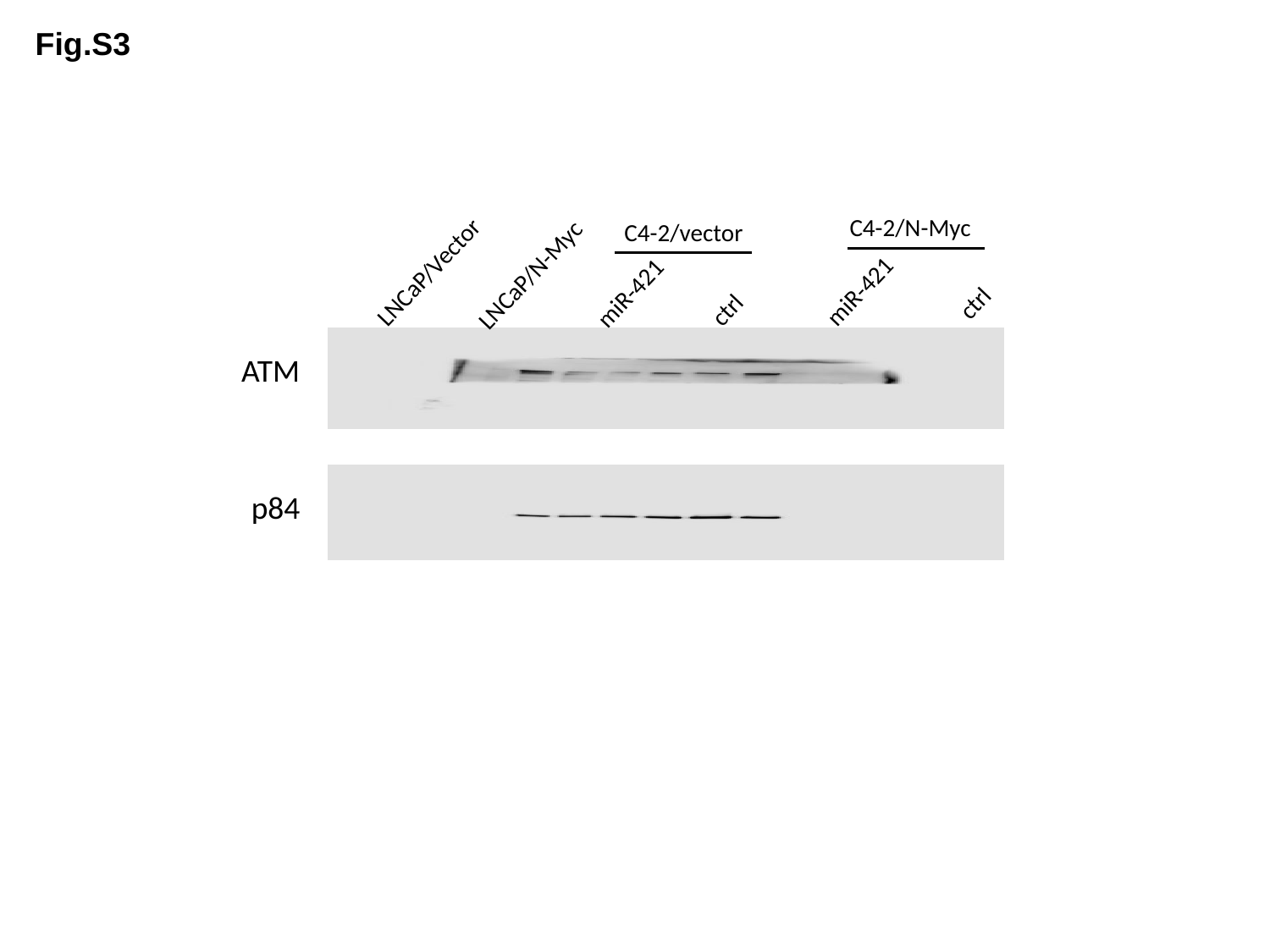

Fig.S3
C4-2/N-Myc
C4-2/vector
LNCaP/Vector
LNCaP/N-Myc
ctrl
ctrl
miR-421
miR-421
ATM
p84

Supplement: Supplementary file 3 — Figure S3. Immunoblot showed that ATM expression was suppressed by overexpressing lentiviral miR-421 in both C4–2/vector and C4–2/N-Myc cells. p84 was used as a loading control. (PPTX 308 kb) [file 12943_2019_941_MOESM3_ESM.pptx]
