# Supplementary material for: N-Myc promotes therapeutic resistance development of neuroendocrine prostate cancer by differentially regulating miR-421/ATM pathway
Source: Mol Cancer. 2019 Jan 18;18:11. doi: 10.1186/s12943-019-0941-2 (PMC6337850; doi:10.1186/s12943-019-0941-2)
Supplement: Supplementary file 4 — Figure S4. N-Myc overexpression in LNCaP and 22RV1 cells differentially regulates the expression of the same target genes. A subset of gene list has been summarized in four different groups: upregulated in both LNCaP/N-Myc and 22RV1/N-Myc, upregulated in LNCaP/N-Myc but downregulated in 22RV1/N-Myc, downregulated in LNCaP/N-Myc but upregulated in 22RV1/N-Myc and both downregulated in LNCaP/N-Myc and 22RV1/N-Myc. (PPTX 68 kb) [file 12943_2019_941_MOESM4_ESM.pptx]

## Slide 1
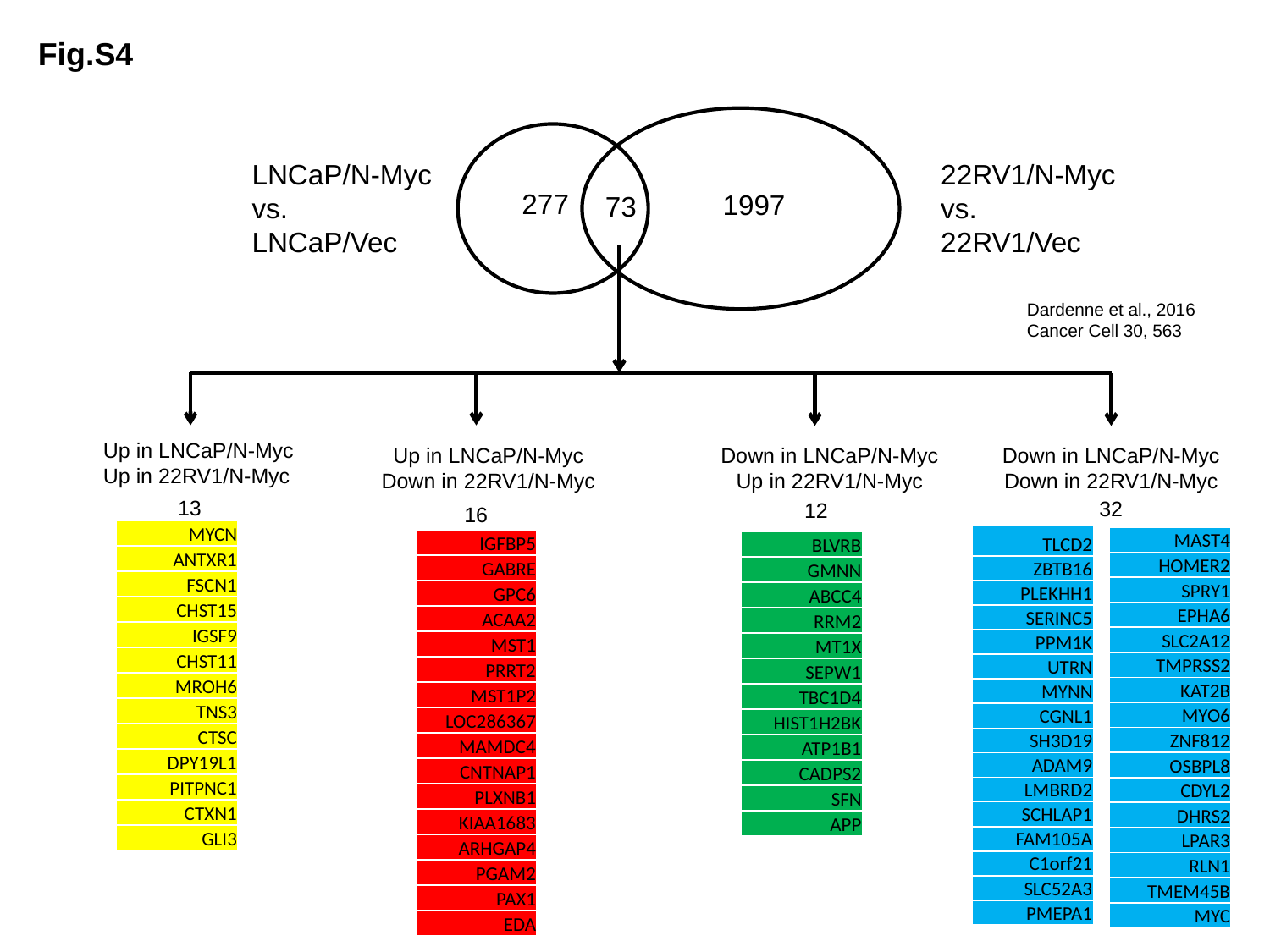

Fig.S4
22RV1/N-Myc
vs.
22RV1/Vec
LNCaP/N-Myc
vs.
LNCaP/Vec
277
1997
73
Dardenne et al., 2016
Cancer Cell 30, 563
Up in LNCaP/N-Myc
Up in 22RV1/N-Myc
Down in LNCaP/N-Myc
Down in 22RV1/N-Myc
Up in LNCaP/N-Myc
Down in 22RV1/N-Myc
Down in LNCaP/N-Myc
Up in 22RV1/N-Myc
13
32
12
16
| MYCN |
| --- |
| ANTXR1 |
| FSCN1 |
| CHST15 |
| IGSF9 |
| CHST11 |
| MROH6 |
| TNS3 |
| CTSC |
| DPY19L1 |
| PITPNC1 |
| CTXN1 |
| GLI3 |
| TLCD2 |
| --- |
| ZBTB16 |
| PLEKHH1 |
| SERINC5 |
| PPM1K |
| UTRN |
| MYNN |
| CGNL1 |
| SH3D19 |
| ADAM9 |
| LMBRD2 |
| SCHLAP1 |
| FAM105A |
| C1orf21 |
| SLC52A3 |
| PMEPA1 |
| MAST4 |
| --- |
| HOMER2 |
| SPRY1 |
| EPHA6 |
| SLC2A12 |
| TMPRSS2 |
| KAT2B |
| MYO6 |
| ZNF812 |
| OSBPL8 |
| CDYL2 |
| DHRS2 |
| LPAR3 |
| RLN1 |
| TMEM45B |
| MYC |
| IGFBP5 |
| --- |
| GABRE |
| GPC6 |
| ACAA2 |
| MST1 |
| PRRT2 |
| MST1P2 |
| LOC286367 |
| MAMDC4 |
| CNTNAP1 |
| PLXNB1 |
| KIAA1683 |
| ARHGAP4 |
| PGAM2 |
| PAX1 |
| EDA |
| BLVRB |
| --- |
| GMNN |
| ABCC4 |
| RRM2 |
| MT1X |
| SEPW1 |
| TBC1D4 |
| HIST1H2BK |
| ATP1B1 |
| CADPS2 |
| SFN |
| APP |
